# Management of Infectious Processes of the Pleural Space: A Review

**DOI:** 10.1155/2012/816502

**Published:** 2012-03-14

**Authors:** Ankur Girdhar, Adil Shujaat, Abubakr Bajwa

**Affiliations:** Division of Pulmonary and Critical Care Medicine, UF College of Medicine at Jacksonville, 655, 8th Street west, Jacksonville, FL 32209, USA

## Abstract

Pleural effusions can present in 40% of patients with pneumonia. Presence of an effusion can complicate the diagnosis as well as the management of infection in lungs and pleural space. There has been an increase in the morbidity and mortality associated with parapneumonic effusions and empyema. This calls for employment of advanced treatment modalities and development of a standardized protocol to manage pleural sepsis early. There has been an increased understanding about the indications and appropriate usage of procedural options at clinicians' disposal.

## 1. Introduction

Any effusion that occurs secondary to an infectious process in the lung parenchyma such as pneumonia or lung abscess is defined as a parapneumonic effusion. A complicated parapneumonic effusion requires an invasive procedure for resolution and usually a bacterial organism can be cultured from the pleural fluid [1]. When a parapneumonic effusion progresses to become frank pus, it is labeled as empyema. Parapneumonic effusion and empyema are both important medical conditions associated with significant morbidity and mortality.

Infection of the pleural space affects approximately 60,000 individuals in the USA annually and has approximately 15% mortality [2]. About 40% of all patients diagnosed with pneumonia have an associated pleural effusion, out of which only a few require active intervention for resolution [1, 3, 4]. Recent epidemiologic studies have indicated that the incidence of empyema has been increasing in the last two decades [5, 6].

In view of the increasing incidence and considerable mortality and morbidity associated with pleural infections, there is a need to utilize modern principles of empyema management that will promote early diagnosis and prompt pleural drainage. It has been observed that any delay in initiating effective drainage can result in prolonged hospital stay, requirement of an invasive procedure for drainage, and further increase in mortality and morbidity [1, 7–9] (Table 1).

## 2. Pathophysiology of a Parapneumonic Effusion

Any inflammation due to an infectious process in the lung parenchyma leads to disturbance in the delicate balance between formation of pleural fluid and its clearance resulting in accumulation of fluid in the pleural space. This pleural fluid initially can be sterile but if left untreated can progress to become an empyema. This progression occurs in three stages (Table 2) [10].

At the very beginning, inflammation due to pneumonia in the lung parenchyma increases vascular as well as visceral pleural membrane permeability by molecules like vascular endothelial growth factor (VEGF) and there is outpouring of inflammatory fluid in the pleural space [11]. This is known as the exudative phase. At this stage, the pleural fluid is nonviscous, free-flowing, and readily drained by thoracentesis or chest tube. During this stage, pleural fluid culture is negative for bacteria, fluid pH is >7.20, the glucose level is within the normal range and lactate dehydrogenase remains <3 times the upper limit of normal [12].

**Figure 1:**
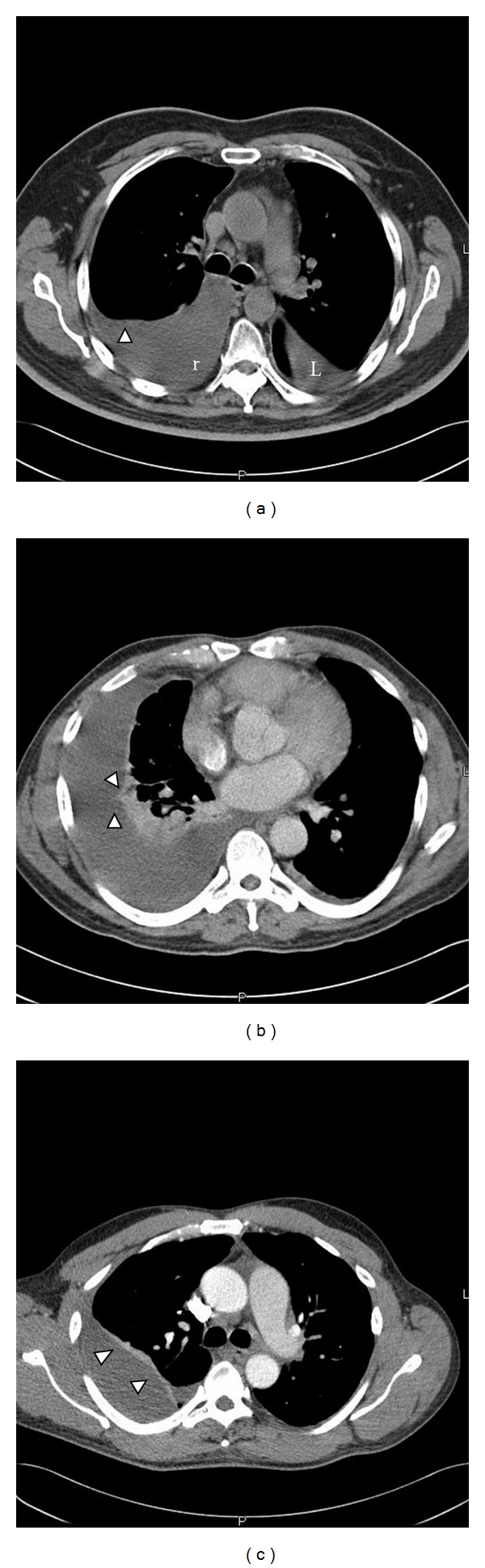
A series of CT images done in patient with parapneumonic effusions. (a) CT image showing a free flowing pleural effusion (r) with a meniscus formation (arrow). There is also some fluid in the fissure on the left side (L). (b) A loculated pleural effusion with loculations seen in the pleural space (arrows). (c) A chronic pleural effusion showing marked pleural thickening (arrows).

If the inflammation proceeds unabated, it leads to purulent and increasingly viscous pleural fluid, which is now rich in inflammatory cytokines like IL-1 and TNF-*α*. IL-1 induces mesothelial cells to release transforming growth factor (TGF-*β*) which is one of the most potent fibrogenic agents ever discovered [13]. This second stage called fibrinopurulent phase is characterized by positive microbial cultures and the effusion now is referred to as “complicated” (Figure 1). Patients with complicated parapneumonic effusions have higher pleural fluid levels of TNF-*α*, which is a marker of the degree of inflammation, than do patients with uncomplicated parapneumonic effusions [14]. Pleural infection during this stage may respond to antibiotics and chest tube drainage but often requires invasive intervention. This is because of the continuing inflammation that there is a deposition of fibrin over the visceral pleura which in turn results in the formation of adhesions that impede lung re-expansion during attempts at fluid drainage. When the pleura is inflamed, the amount of fibrin that is laid down is the result of the balance between fibrinogenesis and fibrinolysis. Fibrogenesis occurs when the factors that favor fibrogenesis such as TNF-*α*, TGF-*β*, and plasminogen activation inhibitor-1 (PAI-1) are dominant. Fibrinolysis occurs when more fibrin is being broken down than is being created [15]. If a fibrinopurulent effusion remains undrained, fibroblasts eventually deposit fibrotic tissue that encases the lung in inelastic peels [16–18]. At this organizing phase, thick pleural peel restricts chest mechanics and often requires surgical decortications to address restrictive impairment.

## 3. Bacteriology

The bacteria isolated from infected pleural effusion vary significantly between community- and hospital-acquired infections. Maskell et al. conducted a large prospective MIST 1 trial (Multicenter Intrapleural Sepsis Trial 1) in 2005 [19]. In their study, 430 subjects were enrolled from 52 centres in the United Kingdom. Positive pleural cultures were found in 232 (54%) of the subjects. The most common pathogen isolated was *Streptococcus milleri* group (29%), followed by *staphylococci* (21%) and *Streptococcus pneumonia* (16%). Only 15% of effusions had anaerobes. Less common organisms responsible for community-acquired infection include other streptococci, enterobacteria, *Haemophilus influenzae*, *Pseudomonas* spp., tuberculosis, and Nocardia. In an earlier study [20], it was reported that nosocomial pleural infections were most commonly caused by methicillin-resistant *Staphylococcus aureus* (27%), other staphylococci (22%) and enterobacteria (20%). Similar results were seen in a recent study of empyema in the intensive care unit setting by Tu et al. [21]. They found that *Klebsiella pneumoniae* was the most isolated microbe and also there was a high prevalence of polymicrobial infection. Even though the MIST 1 trial showed a low incidence of anaerobic organisms causing pleural infections, it is well known that they are difficult to isolate by culture of fluid and/or blood. Previous studies have shown that anaerobic bacteria were cultured in 36 to 76 percent of human empyemas [22, 23] with predominant organisms isolated being *Fusobacterium nucleatum*, *Prevotella* sp, Peptostreptococcus, and the *Bacteroides fragilis* group although B. fragilis is relatively rare [22, 24, 25].

## 4. Therapeutic Approaches to Manage Pleural Infections

There are very few randomized trials regarding management of pleural infections. This limits the evidence base to small observational reports and expert opinions leading to considerable variation in the treatment of individual patients. Depending on institutional expertise, the management of pleural infections can range from noninvasive treatment such as observation and antibiotic therapy to aggressive as well as invasive procedures like therapeutic aspiration, tube thoracostomy and intrapleural fibrinolytics, thoracoscopy, thoracotomy, or open drainage [12].

In recent times, application of these treatment modalities has been greatly aided by advanced imaging studies. With various imaging as well as treatment options at our disposal, there is a need for development of a multidisciplinary approach that can coordinate pulmonary, thoracic surgery, and interventional radiology expertise.

### 4.1. Antibiotics

Almost all patients with parapneumonic effusion will need antibiotic coverage. This coverage can be to treat the pneumonia or empirical coverage for a suspected pleural sepsis [1]. Even if the pleural fluid cultures are negative and there is a strong suspicion of pleural infection, clinician should initiate an empiric anaerobic coverage as an anaerobic infection will not grow well on culture media. According to the bacteriology listed for a community-acquired infection before the first choice will include intravenous amoxicillin with clavulanic acid or a combination of a second-generation cephalosporin (e.g., cefuroxime) and metronidazole or clindamycin if patient is penicillin allergic [26] patients with nosocomial empyema need adequate Gram-negative coverage, as Gram-negative infections are more common in nosocomial empyemas. Coverage should include at least a carbapenem or an antipseudomonal penicillin (e.g., piperacillin/tazobactam), or third- or fourth-generation cephalosporins (e.g., ceftazidime, cefepime) with metronidazole. If there is a suspicion for MRSA, coinfection vancomycin or linezolid can be added. The single exception is that aminoglycosides may be inactivated at low pleural fluid pH [27].

### 4.2. Serial Thoracentesis

Therapeutic thoracentesis has been used for the treatment of parapneumonic effusions for almost two centuries [28]. In recent times, treating empyema or complicated parapneumonic effusions with serial therapeutic pleural aspirations has been largely abandoned. There have been no controlled studies comparing therapeutic thoracentesis with small-tube thoracostomy in the treatment of patients with complicated nonloculated parapneumonic effusions. Most of the recommendations are from some centers [29, 30] who advocate that patients should have daily therapeutic thoracentesis with or without pleural lavage in case of recurrence of infected effusions after initial thoracentesis to allow the pleural fluid to freely flow without any formation of locules until antibiotics resolve the infection. This approach may require an average of eight thoracentesis in >2 to 4 weeks. This was shown in a recent study done by Simmers et al. [31] in which they were able to successfully treat 24 of 29 patients with parapneumonic effusions by means of alternate-day ultrasound-guided pleural aspirations. This approach required that the patients undergo an average of 7.7 ± 3.5 thoracentesis with an average hospitalization of 31 days.

### 4.3. Chest Tube Drainage

Current indications for chest tube drainage are the aspiration of frankly purulent pleural fluid, the identification of organisms on pleural fluid Gram stain or culture, or a pleural fluid pH < 7.2 in the clinical setting of a pneumonic illness [32]. As an exception in a very large simple parapneumonic effusion, chest tube drainage may be done for symptomatic relief. According to old literature, chest tube drainage is most commonly achieved by a standard (24–28 french) intercostal chest drain, that is, positioned in the dependent part of a free-flowing pleural effusion (most often the posterior costophrenic recess). Using an imaging modality like ultrasound for inserting chest tube is advised as thickened parietal pleura, adhesions, or loculations often complicate insertion. Complete re-expansion of the lung, as demonstrated by repeat imaging, resolution of clinical and laboratory signs of infection, and avoidance of surgical drainage, defines successful drainage.

**Figure 2:**
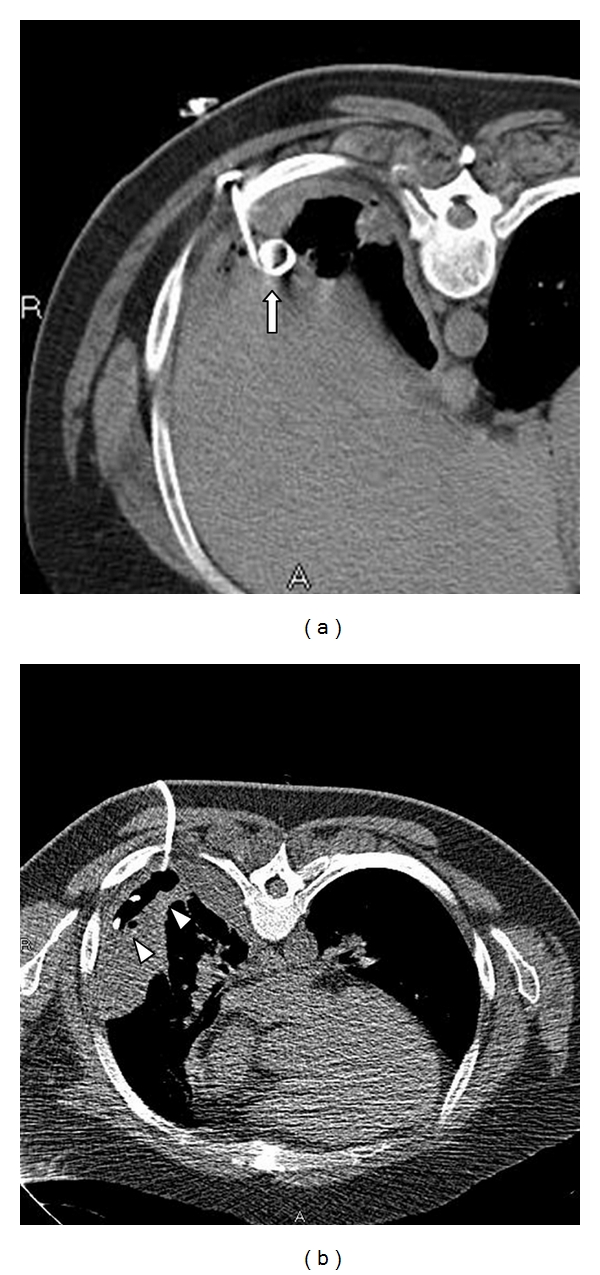
CT images after chest tube drainage. (a) Image shows placement of pigtail catheter (arrow) in the posterior recess confirmed with CT. (b) Placement of small-bore pigtail catheter (arrowheads) in the small loculated effusion with the help of CT guidance.

Till recent times, the common thinking was that smaller bore chest drains are likely to fail in the presence of pus with a high viscosity. However, some prospective studies [33–35] have found that 8- to 12-french pigtail catheters or 10- to 14-french catheters inserted with the Seldinger technique under US or CT guidance (Figure 2) were at least as effective as larger catheters inserted without imaging. Occlusion of the smaller drains can be avoided by the use of suction (20 cm H_2_O) and regular flushes (e.g., 30 mL normal saline every 6 hours). If the patient has not demonstrated significant improvement within 24 h of initiating tube thoracostomy, either the pleural drainage is unsatisfactory or the patient is receiving the wrong antibiotics. Unsatisfactory pleural drainage can be due to the tube being in the wrong location, loculation of the pleural fluid, or a fibrinous coating of the visceral pleura, which prevents the underlying lung from expanding. If drainage is inadequate, ultrasonography or a CT scan should be obtained to delineate which of the above factors is responsible. Data is still lacking to define the right time to remove the chest drain and thus general recommendations are to remove the drain when the daily output falls to less than 150 cc for 2 consecutive days, in the setting of clinical and radiographic improvement.

The evidence base developing for small bore drains estimates a failure rate of 19% with their use in draining empyema [36]. A very recent study [35] of 71 complicated parapneumonic effusions and 70 empyemas drained with ultrasonographically guided small catheters showed a success rate of 80% (48/60) when the initial ultrasonography did not reveal significant loculations. In those patients with a complex septated pattern on ultrasonography, the success rate was still 51% (41/81). Authors concluded that the threshold for using fibrinolytics and large-bore catheters should be low in empyema.

**Figure 3:**
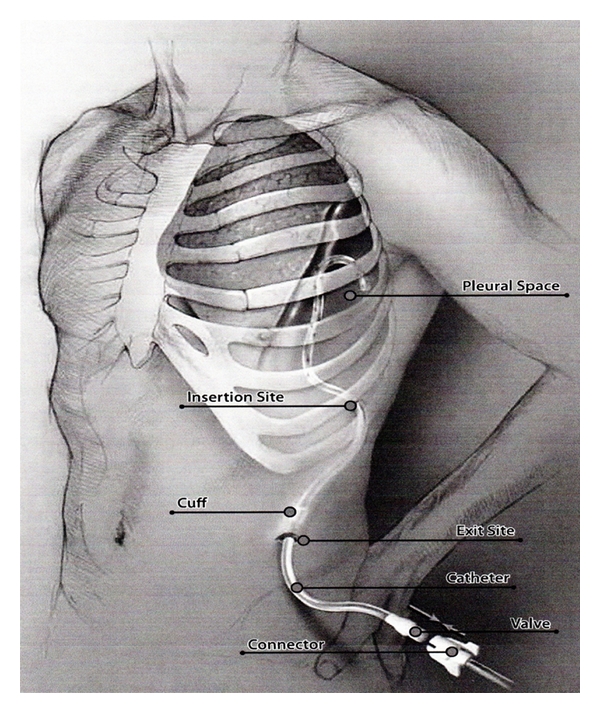
A pictorial representation of a chronic indwelling catheter (Aspira) which is tunneled beneath the skin to enter the pleural cavity at a distant site. This assembly prevents introduction of infection in the pleural cavity and can provide long term drainage of infected pleural effusion.

Long-term indwelling catheters (Figure 3) are being increasingly used to drain malignant pleural effusions. Development of infection in the pleural space has been cited as a complication of this product. An article was published in 2008 [37] with two reports of use of indwelling catheters to treat pleural infection. The first case had a persistent bronchopleural fistula and the second case had esophageal rupture due to necrotizing TB lymphadenitis resulting in development of empyema in both cases. These cases suggested that small-bore indwelling catheters can have as successful outcomes as open drainage procedures and in addition provide patients with better quality of life during sustained pleural drainage. The current understanding is that during the early phase of pleural infection, short-term fine-bore pigtail catheter drainage can be useful, while for chronic pleural infection, long-term drainage can be effective without the problems of catheter blockage or tract infection. This approach needs validation with larger patient samples and randomized trials.

### 4.4. Intrapleural Fibrinolytics and DNase

Drainage of pleural fluid becomes challenging when there is formation of loculations inside the pleural cavity which resist drainage with a single chest tube. This has generated considerable interest in the use of intrapleural fibrinolytic agents and DNase (Table 3), which may facilitate fluid drainage by dissolving fibrinous adhesions. Development of dense layers of fibrin and loculations in a complicated parapneumonic effusions and empyemas are as a result of the procoagulant state within the pleural space as discussed in the pathophysiology of pleural infections. It, therefore, seems highly plausible that intrapleural fibrinolytics given early in the fibrinopurulent phase should prevent loculations and promote pleural drainage. Small studies [38–41] have reported the beneficial effects of therapy with streptokinase, urokinase, and rtPA for avoiding surgery and improving the radiographic appearance of loculated effusions. Based on these early reports of efficacy from smaller studies, the BTS [26, 42] and the ACCP [7] (Table 1) guidelines have recommended fibrinolytic drugs as possible management options.

Till date, the largest randomized control trial of fibrinolytic therapy is the Multicenter Intrapleural Sepsis Trial (MIST1) done by Maskell et al. [19]. Study centers in this trial placed small-bore chest tubes (median size, 12F) without image guidance in 427 patients with complicated parapneumonic effusions (pleural fluid pH < 7.20, with signs of infection, or positive findings from a pleural fluid Gram stain or culture) or frank empyema and instilled streptokinase or placebo. The trial observed no benefits from streptokinase administration in terms of survival, decreased hospital stay, or need for surgery. However, there was a criticism about the methodology and implementation of this trial [43–45]. Patients did not undergo CT scanning or US imaging to identify locules or place chest tubes, and correct tube positioning was not confirmed after placement. There were concerns about the generalization of findings as no standardized protocols were used across the 52 centers to direct antibiotic or other treatments or to select patients who had not responded to fibrinolysis for surgery. Many of these centers lacked on-site surgical expertise and contributed only small numbers of patients. Even the drainage techniques were questioned as study design permitted small-bore chest tubes but did not report on pleural drainage volumes. Furthermore, streptokinase was mailed to study centers after randomization, which delayed fibrinolysis. Mortality as one of the endpoints was doubted as patients with serious concomitant illnesses that made survival beyond three months unlikely were excluded from the study. It was speculated that use of intrapleural streptokinase might yield better results in improving short-term mortality in a carefully selected patient population [43]. These deficiencies do not invalidate this large randomized trial, but concerns remain about the validity of its results with regards to younger, more severely ill patients and in different health care settings.

Streptokinase often loses effectiveness due to immune-mediated neutralization; therefore, studies [40, 46, 47] have been done using rtPA as the primary fibrinolytic. These studies estimate success rate of 86% with rtPA.

Similar results as in MIST1 were found in a meta-analysis [48] done subsequently to evaluate the benefit of fibrinolytic therapy in pleural sepsis. A Cochrane review [49] that included some studies (*n* = 761) also failed to show a reduction in death among patients who received fibrinolytic therapy (28 versus 33 percent). In view of conflicting results in different studies, currently there is not enough evidence to support routine fibrinolytic therapy for every patient with parapneumonic effusions.

Deoxyribose nucleoprotein content plays a major role in increasing the viscosity of pus in the pleural space. Intrapleural fibrinolytics have negligible effects on decreasing the viscosity of empyema pus in contrast to agents that depolymerize DNA, such as human recombinant deoxyribonuclease. Benefit of intrapleural human recombinant DNase in the treatment of empyema following failure of streptokinase has been reported only in case reports [50]. In a recent UK trial comparing the effects of intrapleural tPA, intrapleural fibrinolytics and both combined with placebo showed insignificant response in pleural infection resolution with tPA or DNase alone. On the other hand, the combination of tPA-DNase instilled in the intrapleural space improved fluid drainage and reduced the frequency of surgical referral and the duration of the hospital stay [51]. These initial case reports and trial hint toward a potential new therapy which can improve outcomes of semi-invasive therapies.

### 4.5. Thoracoscopy

Thoracoscopy is a technique which is able to provide a minimally invasive access to the pleural space to suction viscous pleural fluid, lyse adhesion in loculated pleural effusions, and place chest tubes in dependent regions of pleural fluid under direct visualization [12]. Loculations can be broken down, the visible pleural space completely drained, and an intercostal chest tube can be optimally placed [12]. Thoracoscopy in comparison to thoracostomy has the advantage of having less postoperative pain, lower costs, shorter hospital stays, and better cosmetic results [52]. Available thoracoscopic procedures include medical thoracoscopy and video-assisted thoracoscopic surgery (V-ATS).

**Figure 4:**
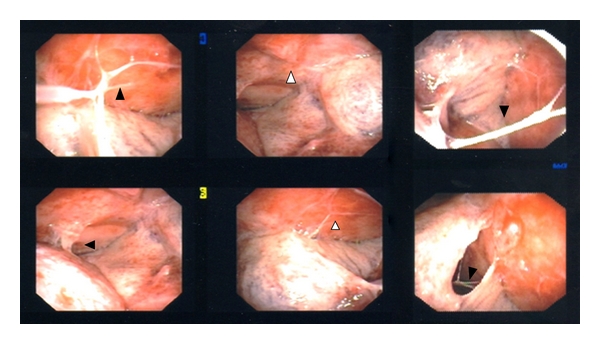
Thoracoscopic views of a complicated parapneumonic effusion. Multiple pleural adhesions (black arrowheads) are seen which prevent lungs from re-expanding. There are also seen inflamed pleura (white arrowheads) which represent nonresolving infection.

Medical thoracoscopy (Figure 4) has been shown to provide resolution of tuberculous pleural effusions by repeated adhesionolysis since early part of 20th century in Europe [53, 54]. Medical thoracoscopy is a cheap and quick procedure which can easily be done in an endoscopy suit with patient under conscious sedation and breathing spontaneously within 30–60 minutes [55]. Medical thoracoscopy is performed via single chest port in contrast to VATS, and does not require complete collapse of the lung. Limitation of medical thoracoscopy lies in its inability to fully examine the pleural cavity and to perform pleurectomy if needed. Additionally, debridement done using medical thoracoscopy is time consuming and cumbersome.

Again, there has been a lack of large randomized controlled trial for establishing the role of medical thoracoscopy. A recent case series [56] which analyzed the benefit of medical thoracoscopy for treatment of ultrasonographically stratified multiloculated pleural effusion showed a primary success rate of 91%. Taking in account patients who required additional chest tube insertion or second medical thoracoscopy procedure, the success rates further improved to 94%. 6% of cases required conversion to open drainage. This case series reported the use of intrapleural fibrinolytics as an adjunctive therapy after the thoracoscopy procedure in 49% of cases. Complications occurred in 9% of patients with no mortality observed due to the procedure itself.

VATS is a procedure which is performed by a cardio-thoracic surgeon generally using a three-entry port and a double-lumen endotracheal tube. Using VATS, surgeons can also perform decortication and pleurectomy if needed. Even though VATS in comparison to medical thoracoscopy can provide the operator with a much larger access to the pleural space, it may still prove out to be inadequate to treat thick empyemas complicated by dense adhesions and multiple loculations. Studies on VATS procedure have reported a success rate of 60–100%. Currently, VATS is reserved for treating complicated fibrinopurulent effusions, with some surgeons using it during the organizing phase and then converting to thoracostomy if it fails [57–60].

### 4.6. Open Drainage

An open drainage procedure is employed when the minimally invasive procedures fail to achieve acceptable resolution, defined as re-expansion of lung to the chest wall. In the early exudative or fibrinopurulent stages, an open drainage procedure helps to control the pleural sepsis while the main aim in an organizing phase is to remove the fibrotic peel that encases the lung in order to help it to re-expand and improve chest dynamics [12, 61, 62].

Open drainage is achieved using two types of approaches. First being thoracotomy with drainage and subsequent closure of the chest with one or more drains left in the pleural cavity. Second approach involves creating a window in the pleural cavity by chest wall incision and rib resection, which provides continuous drainage of the chest cavity. This is called thoracostomy. Through the window in the chest wall drainage can be facilitated by inserting chest tubes. After complete removal of the empyema, chest tubes can be withdrawn. Thoracotomy procedure can also help in complete or partial decortication of the pleural membranes coated with fibrous tissue which will in turn expedite evacuation of thick pus in the pleural cavity and let the lung re-expand [63]. Debridement in comparison to decortication which is a major thoracic operation is less aggressive and can be better tolerated by patients who are markedly debilitated [64].

In a review of 25 patients [65] who underwent either decortication or debridement for empyema drainage, the outcomes were studied by measuring the change of the pleural cavity size before, immediately after surgery, and on followup. On followup imaging, the eventual size of the pleural cavity was not different between the two procedure groups (*P* < 0.937). Thus, almost similar results were achieved by debridement alone without decortication in patients presenting with empyema, despite the presence of an underlying trapped lung.

## 5. Conclusions

**Figure 5:**
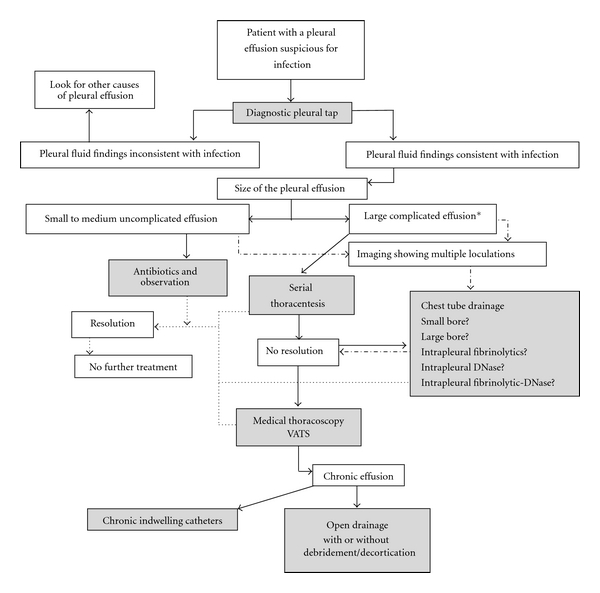
A schematic flow chart summarizing the various treatment modalities available for managing pleural infection and various stages where each of them may be used. Decisions regarding timing of each treatment option may vary according to institutional expertise. *Empyema or effusions with either gram stain or culture positive, pH < 7.2, glucose < 60 mg/dL, LDH < 1000.

The management principles for pleural infection have come a long way from employing antibiotic therapy and thoracentesis to the current availability of semi-invasive and invasive procedures. The key to successful management of pleural infection still remains to be early diagnosis and initiation of treatment. Due to the paucity of robust clinical trials, the treatment modality or the management approach chosen largely depends on individual and institutional expertise. Clinicians are encouraged to develop standardized protocols using best practices reported in the literature, for early identification and management (Figure 5).

Use of advanced imaging like ultrasound and CT scans widens the scope of diagnosing and treating effusions seen on a routine postero-anterior chest radiograph. Observation is usually adequate for a small (<10 mm) unseptated, free flowing effusions. Any other effusions warrant a diagnostic thoracentesis. If the aspirated fluid fulfills the criteria for being infected (pH < 7.2, glucose < 40 mg/dL, culture positive), a prompt plan for its drainage is needed. Currently, large bore tube thoracostomy is the treatment option of choice for patients with empyema, but data is accumulating for treating parapneumonic effusions with small-bore intercostal drains.

The use of fibrinolytics still remains controversial. Fibrinolytics will have more defined role for treating loculated parapneumonic effusions and empyema, particularly in young, acutely ill patients, poor surgical candidates, and in centres with inadequate surgical facilities. Early thoracoscopy is an alternative to thrombolytics. Local expertise will dictate the choice between therapeutic thoracentesis, intrapleural fibrinolytics, and medical thoracoscopy as well as conversion to open drainage when thoracoscopy fails till randomized trials provide with better evidence.

## Figures and Tables

**Table 1:** Pleural infections staging and recommended drainage [7].

Category	Pleural space anatomy		Pleural fluid chemistry	Risk of poor outcome	Drainage
1	Minimal free-flowing effusion (<10 mm on lateral decubitus)	and	Gram stain and culture results unknown	Very low	No
2	Small to moderate free-flowing effusion (≥10 mm and less than one half hemithorax)	and	Negative Gram stain and culture	Low	No
3	Large, free-flowing effusion (≥ one half hemithorax), loculated effusion, or effusion with thickened parietal pleura	or	Positive Gram stain and/or culture	Moderate	Yes
4	Empyema		pus	High	Yes

**Table 2:** Different stages in the evolution of an infected pleural effusion with associated pathological changes and pleural fluid findings.

Phase	Pathology	Pleural fluid findings
Exudative	Increased permeability of vascular and visceral pleural membranes VEGF	Nonviscous
		Free flowing
		Readily drained
		Pleural fluid Cx negative

Fibrinopurulent	Fibrin deposition on visceral pleura Locules formation IL-8, TNF-*α*	pH > 7.20
		Glucose within normal ranges
		LDH < 3 times ULN
		Viscous
		More viscous
		Pleural fluid cx positive
		Typical “complicated” effusion

Organizing	Fibroblast entry Pleural peel TGF-*β*	Thick pus
		Very viscous
		pH < 7.20
		Glucose < 40
		LDH > 3 times ULN

LDH: lactate dehydrogenase.

ULN: upper limits of normal.

VEGF: vascular endothelial growth factor.

IL-8: interleukin 8.

TNF-*α*: tumor necrosis factor-alpha.

TGF-*β*: transforming growth factor-beta.

**Table 3:** Various intrapleural fibrinolytics (Adapted from Colice et al. [7]).

Fibrinolytic	Dose	Instillation	Duration
Streptokinase	250,000 IU	100–200 cc NS	QD for up to 7 days
Urokinase	10,000 IU	100 cc NS	QD for up to 3 days
t-PA	10–25 mg	100 cc NS	BID for up to 5 days

t-PA: tissue plasminogen activator.

IU: international units.

NS: normal saline.

QD: every day.

BID: twice a day.

## References

[1] Chapman SJ, Davies RJO (2004). Recent advances in parapneumonic effusion and empyema. *Current Opinion in Pulmonary Medicine*.

[2] Sahn SA (1993). Management of complicated parapneumonic effusions. *American Review of Respiratory Disease*.

[3] Davies CWH, Kearney SE, Gleeson FV, Davies RJO (1999). Predictors of outcome and long-term survival in patients with pleural infection. *American Journal of Respiratory and Critical Care Medicine*.

[4] Mandal AK, Thadepalli H, Mandal AK, Chettipally U (1998). Outcome of primary empyema thoracis: therapeutic and microbiologic aspects. *Annals of Thoracic Surgery*.

[5] Farjah F, Symons RG, Krishnadasan B, Wood DE, Flum DR (2007). Management of pleural space infections: a population-based analysis. *Journal of Thoracic and Cardiovascular Surgery*.

[6] Finley C, Clifton J, FitzGerald JM, Yee J (2008). Empyema: an increasing concern in Canada. *Canadian Respiratory Journal*.

[7] Colice GL, Curtis A, Deslauriers J (2000). Medical and surgical treatment of parapneumonic effusions: an evidence-based guideline. *Chest*.

[8] Heffner JE, McDonald J, Barbieri C, Klein J (1995). Management of parapneumonic effusions: an analysis of physician practice patterns. *Archives of Surgery*.

[9] Chu MWA, Dewar LRS, Burgess JJ, Busse EGF (2001). Empyema thoracis: lack of awareness results in a prolonged clinical course. *Canadian Journal of Surgery*.

[10] Andrews NC, Parker EF, Shaw RR (1962). Management of nontruberculous empyema. *American Review of Respiratory Disease *.

[11] Grove CS, Lee YCG (2002). Vascular endothelial growth factor: the key mediator in pleural effusion formation. *Current Opinion in Pulmonary Medicine*.

[12] Light RW (2006). Parapneumonic effusions and empyema. *Proceedings of the American Thoracic Society*.

[13] Chung CL, Chen CH, Sheu JR, Chen YC, Chang SC (2005). Proinflammatory cytokines, transforming growth factor-*β*1, and fibrinolytic enzymes in loculated and free-flowing pleural exudates. *Chest*.

[14] Alexandrakis MG, Coulocheri SA, Bouros D, Eliopoulos GD (1999). Evaluation of ferritin, interleukin-6, interleukin-8 and tumor necrosis factor alpha in the differentiation of exudates and transudates in pleural effusions. *Anticancer Research*.

[15] Idell S, Zwieb C, Kumar A, Koenig KB, Johnson AR (1992). Pathways of fibrin turnover of human pleural mesothelial cells in vitro. *American Journal of Respiratory Cell and Molecular Biology*.

[16] Idell S, Girard W, Koenig KB, McLarty J, Fair DS (1991). Abnormalities of pathways of fibrin turnover in the human pleural space. *American Review of Respiratory Disease*.

[17] Alemán C, Alegre J, Monasterio J (2003). Association between inflammatory mediators and the fibrinolysis system in infectious pleural effusions. *Clinical Science*.

[18] Sasse SA, Jadus MR, Kukes GD (2003). Pleural fluid transforming growth factor-*β*
_1_ correlates with pleural fibrosis in experimental empyema. *American Journal of Respiratory and Critical Care Medicine*.

[19] Maskell NA, Davies CWH, Nunn AJ (2005). U.K. controlled trial of intrapleural streptokinase for pleural infection. *New England Journal of Medicine*.

[20] Maskell NA, Davies CW, Jones E, Davies RJO The characteristics of 300 patients participating in the MRC/BTS multicenter intra-pleural streptokinase vs. placebo trial (ISRCTN-39138989).

[21] Tu CY, Hsu WH, Hsia TC (2006). The changing pathogens of complicated parapneumonic effusions or empyemas in a medical intensive care unit. *Intensive Care Medicine*.

[22] Brook I, Frazier EH (1993). Aerobic and anaerobic microbiology of empyema: a retrospective review in two military hospitals. *Chest*.

[23] Bartlett JG, Gorbach SL, Thadepalli H, Finegold SM (1974). Bacteriology of empyema. *The Lancet*.

[24] Civen R, Jousimies-Somer H, Marina M, Borenstein L, Shah H, Finegold SM (1995). A retrospective review of cases of anaerobic empyema and update of bacteriology. *Clinical Infectious Diseases*.

[25] Boyanova L, Gergova G, Iotov D (2004). Anaerobic microbiology in 198 cases of pleural empyema: a Bulgarian study. *Anaerobe*.

[26] Davies CWH, Gleeson FV, Davies RJO (2003). BTS Pleural Disease Group, a sub-group of the BTS Standards of Care Committee: BTS guidelines for the management of pleural infection. *Thorax*.

[27] Vaudaux P, Waldvogel FA (1980). Gentamicin inactivation in purulent exudates: role of cell lysis. *Journal of Infectious Diseases*.

[28] Bowditch HI (1853). Paracentesis thoracic: an analysis of 25 cases of pleuritic effusion. *American Medical Monthly*.

[29] Ryaa Storm HK, Krasnik M, Bang K, Frimodt-Moller N (1992). Treatment of pleural empyema secondary to pneumonia: thoracocentesis regimen versus tube drainage. *Thorax*.

[30] Sasse S, Nguyen T, Teixeira LR, Light RW (1999). The utility of daily therapeutic thoracentesis for the treatment of early empyema. *Chest*.

[31] Simmers TA, Jie C, Sie B (1999). Minimally invasive treatment of thoracic empyema. *Thoracic and Cardiovascular Surgeon*.

[32] Heffner JE, Brown LK, Barbieri C, DeLeo JM (1995). Pleural fluid chemical analysis in parapneumonic effusions: a meta-analysis. *American Journal of Respiratory and Critical Care Medicine*.

[33] Shankar S, Gulati M, Kang M, Gupta S, Suri S (2000). Image-guided percutaneous drainage of thoracic empyema: can sonography predict the outcome?. *European Radiology*.

[34] Ali I, Unruh H (1990). Management of empyema thoracis. *Annals of Thoracic Surgery*.

[35] Ashbaugh DG (1991). Empyema thoracis: factors influencing morbidity and mortality. *Chest*.

[36] Keeling AN, Leong S, Logan PM, Lee MJ (2008). Empyema and effusion: outcome of image-guided small-bore catheter drainage. *CardioVascular and Interventional Radiology*.

[37] Davies HE, Rahman NM, Parker RJ, Davies RJO (2008). Use of indwelling pleural catheters for chronic pleural infection. *Chest*.

[38] Diacon AH, Theron J, Schuurmans MM, Van De Wal BW, Bolliger CT (2004). Intrapleural streptokinase for empyema and complicated parapneumonic effusions. *American Journal of Respiratory and Critical Care Medicine*.

[39] Bouros D, Schiza S, Tzanakis N, Chalkiadakis G, Drositis J, Siafakas N (1999). Intrapleural urokinase versus normal saline in the treatment of complicated parapneumonic effusions and empyema: a randomized, double-blind study. *American Journal of Respiratory and Critical Care Medicine*.

[40] Gervais DA, Levis DA, Hahn PF, Uppot RN, Arellano RS, Mueller PR (2008). Adjunctive intrapleural tissue plasminogen activator administered via chest tubes placed with imaging guidance: effectiveness and risk for hemorrhage. *Radiology*.

[41] Davies RJO, Traill ZC, Gleeson FV (1997). Randomised controlled trial of intrapleural streptokinase in community acquired pleural infection. *Thorax*.

[42] Balfour-Lynn IM, Abrahamson E, Cohen G (2005). BTS guidelines for the management of pleural infection in children. *Thorax*.

[43] Heffner JE (2005). Multicenter trials of treatment for empyema—after all these years. *New England Journal of Medicine*.

[44] Diacon AH, Koegelenberg CFN, Bolliger CT (2005). A trial of intrapleural streptokinase. *New England Journal of Medicine*.

[45] Bouros D, Antoniou KM, Light RW (2006). Intrapleural streptokinase for pleural infection. *British Medical Journal*.

[46] Walker CA, Shirk MB, Tschampel MM, Visconti JA, Morand BR, Guévremont C (2003). Intrapleural alteplase in a patient with complicated pleural effusion. *Annals of Pharmacotherapy*.

[47] Skeete DA, Rutherford EJ, Schlidt SA, Abrams JE, Parker LA, Rich PB (2004). Intrapleural tissue plasminogen activator for complicated pleural effusions. *Journal of Trauma*.

[48] Tokuda Y, Matsushima D, Stein GH, Miyagi S (2006). Intrapleural fibrinolytic agents for empyema and complicated parapneumonic effusions: a meta-analysis. *Chest*.

[49] Cameron R, Davies HR (2008). Intra-pleural fibrinolytic therapy versus conservative management in the treatment of adult parapneumonic effusions and empyema. *Cochrane Database of Systematic Reviews*.

[50] Simpson G, Roomes D, Heron M (2000). Effects of streptokinase and deoxyribonuclease on viscosity of human surgical and empyema pus. *Chest*.

[51] Rahman NM, Maskell NA, West A (2011). Intrapleural use of tissue plasminogen activator and DNase in pleural infection. *New England Journal of Medicine*.

[52] Roberts JR, Weiman DS, Miller DL, Afifi AY, Kraeger RR (2003). Minimally invasive surgery in the treatment of empyema: intraoperative decision making. *Annals of Thoracic Surgery*.

[53] Tschopp JM, Boutin C, Astoul P (2002). Talcage by medical thoracoscopy for primary spontaneous pneumothorax is more cost-effective than drainage: a randomised study. *European Respiratory Journal*.

[54] Tschopp JM, Brutsche M, Frey JG (1997). Treatment of complicated spontaneous pneumothorax by simple talc pleurodesis under thoracoscopy and local anaesthesia. *Thorax*.

[55] Loddenkemper R (1998). Thoracoscopy—state of the art. *European Respiratory Journal*.

[56] Brutsche MH, Tassi GF, Györik S (2005). Treatment of sonographically stratified multiloculated thoracic empyema by medical thoracoscopy. *Chest*.

[57] Tassi GF, Davies RJO, Noppen M (2006). Advanced techniques in medical thoracoscopy. *European Respiratory Journal*.

[58] Drain AJ, Ferguson JI, Sayeed R, Wilkinson S, Ritchie A (2007). Definitive management of advanced empyema by two-window video-assisted surgery. *Asian Cardiovascular and Thoracic Annals*.

[59] Waller DA (2002). Thoracoscopy in management of postpneumonic pleural infections. *Current Opinion in Pulmonary Medicine*.

[60] Cassina PC, Hauser M, Hillejan L, Greschuchna D, Stamatis G, Deslauriers J (1999). Video-assisted thoracoscopy in the treatment of pleural empyema: stage-based management and outcome. *Journal of Thoracic and Cardiovascular Surgery*.

[61] Sasse SA (1996). Parapneumonic effusions and empyema. *Current Opinion in Pulmonary Medicine*.

[62] Thurer RJ (1996). Decortication in thoracic empyema: indications and surgical technique. *Chest Surgery Clinics of North America*.

[63] Mandal AK, Thadepalli H, Mandal AK, Chettipally U (1998). Outcome of primary empyema thoracis: therapeutic and microbiologic aspects. *Annals of Thoracic Surgery*.

[64] Pothula V, Krellenstein DJ (1994). Early aggressive surgical management of parapneumonic empyemas. *Chest*.

[65] Mackinlay TAA, Lyons GA, Chimondeguy DJ, Barboza Piedras MA, Angaramo G, Emery J (1996). VATS debridement versus thoracotomy in the treatment of loculated postpneumonia empyema. *Annals of Thoracic Surgery*.

